# Determining the sequence of extracting *Pinus radiata* bark to maximize the total yield of extractives–towards the development of a bark-based biorefinery

**DOI:** 10.1186/s40643-025-00896-3

**Published:** 2025-05-31

**Authors:** Sumanth Ranganathan, Sylke H. Campion, Reid A. Dale, Queenie L. Tanjay, Rachel H. Murray, Anna de Lena, Michael Robertson, Armin Thumm, Mark West

**Affiliations:** Scion, 49 Sala Street, Rotorua, 3010 New Zealand

**Keywords:** *Pinus radiata*, Bark biorefinery, Sequential extraction, Extractives, Hydrophilic, Lipophilic, Circular bioeconomy

## Abstract

**Abstract:**

*Pinus radiata* is the dominant tree species in exotic plantation forestry of New Zealand producing timber for construction and pulp and paper. Additionally, the processing yields large amounts of bark as a byproduct that is either left at the harvest site or used for landscaping. *P. radiata* bark is rich in biochemical extractives containing polyphenols and waxes on sequential extraction with hydrophilic and lipophilic solvents, respectively. Previous studies have exclusively focussed on the effect of parameters such as solvent type, bark to solvent ratio, and extraction time on the yield of extractives. However, two parameters were always maintained constant: solvent order (lipophilic to hydrophobic) and particle size. This work investigated the the combined impact of these two parameters on total yield and product quality by using two solvents- water and hexane. Total extractives were highest when water was used first (11.74% and 9.45%) compared to hexane (10.53% and 6.53%). The individual yields of hexane extractives were in the range of 2.25–2.9% while those of water were 4.30–9.24%. Chemical analyses of the extracts and residues showed no qualitative differences, indicating the order in which bark is extracted does not alter the extract composition. Moreover, the results have successfully established that extracting bark with water first followed by hexane will increase the total yield of extractives and increasing particle size decreases the total yield of the sequential extraction.

**Graphical Abstract:**

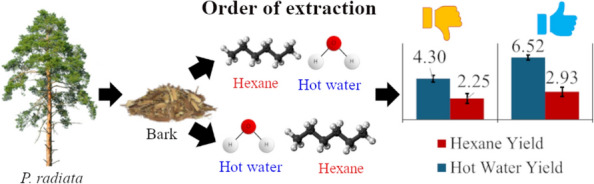

## Introduction

Petrochemical feedstocks have been influential in providing modern luxuries such as chemicals, energy, and materials. However, issues such as waste accumulation, environmental pollution, climate change, and resource depletion are common side-effects of this technology. With a growing population and its associated demand, there is an urgent need to switch to a cleaner means of production, whilst maintaining similar or better benefits than existing technology (Feng et al. 2013; Neiva et al. 2018, 2020). One potential replacement is lignocellulosic biomass that is abundant and often underutilised. Tree bark is a good example of lignocellulosic biomass that is a complex and heterogeneous outer layer making up approximately 9–15% (weight) of the tree (Chen et al. 2020). Globally, ca. 400 million tons of this ‘waste’ residue are produced from forest harvest each year. However, only a fraction of this is currently being utilized, primarily for landscaping or for energy; the rest is left at the harvest or handling site to naturally decompose. Tree bark is rich in chemicals comprising of primary (mainly polymers such as cellulose, hemicellulose, and lignin) and secondary metabolites (often referred to as extractives such as tannin, suberin, and terpenoids) with the composition varying widely depending on the type and age of the tree it is harvested from. Given the vast amount of chemicals bark has to offer and the environmental issues the scientific community wishes to solve, valorisation or biorefining technologies to extract components from bark have been suggested as a valuable replacement for platform chemicals derived from fossil resources (Şen et al. 2023; Vangeel et al. 2023; Wijeyekoon et al. 2021). For instance, most of the research so far has focussed on polyphenolic extracts used to replace petrochemically sourced adhesives or polyurethane foams (Dhawale et al. 2022; Feng et al. 2013; Supriyadi et al. 2025). 

The plantation forest sector in New Zealand comprises of both native and exotic trees, with *Pinus radiata* being the predominant species of commercial forests (> 90%). Annually, approximately 38.3 million m^3^ of trees are harvested yielding ca. 2.6 million green ton of bark as a residue. Besides the lignocellulosic backbone and ash content, *P. radiata* bark is also rich in hydrophilic and lipophilic extractives (Bridson et al. 2024; Patel et al. 2024). Hot water extracts of *P. radiata* bark are flavonoid-rich and possess anti-inflammatory, anticarcinogenic, and antioxidant properties. There is also a potential use of these extracts as a termite deterrent and adhesive for particle board manufacture (Mun et al. 2020).The lipophilic extract of P. radiata bark contain terpenes, resin acids, fatty acids and alcohols, in addition to suberin. One potential use of these lipophilic extracts is to replace the petrochemically sourced vaseline in cosmetics such as soaps, creams, and lotions (Sandoval-Rivas et al. 2021). A recent study reported the use of supercritical carbon dioxide extracted lipophilic *P. radiata* bark waxes to impregnate wood for enhancing water repellence (Elustondo et al. 2022). Another report has also detailed the use of suberin from *P. radiata* bark to produce hydrophobic polymers (Quilter et al. 2024). An abundantly available resource rich in chemicals with a multitude of products and application areas make *P. radiata* bark a prime candidate for a bark-based biorefinery. The development of a bark-based biorefinery has already been conceptualised and published for Norway spruce (*Piecea abies*) (Rietzler & Ek 2021; Rietzler et al. 2022), maritime pine (*Pinus pinaster*) (Abilleira et al. 2021), radiata pine (*Pinus radiata*) (Ku et al. 2011; Mun et al. 2020), Scots pine (*Pinus sylvestris*) (Barbini et al. 2021), and silver birch (*Betula pendula*) (Ferreira et al. 2017).

The term “biorefinery” broadly implies the processing of biomass to produce value-added products such as fuels, chemicals, and materials (Conteratto et al. 2021). In other words, a biorefinery mimics a conventional petrochemical refinery, however with key differences being feedstock, building block content, processes involved, and intermediates produced, to name a few. Most biomass feedstocks are highly heterogeneous and need to be separated into their constituent components for use as platform chemicals. In a biorefinery, this can be achieved through extraction processes using a solvent (extractant), as opposed to temperature-based separation in a traditional petrochemical refinery, which makes the design of a commercial biorefining facility quite challenging (Blair et al. 2017; de Jong & Jungmeier 2015). The yield and product quality of a biorefinery, particularly the extracts, will depend on the choice of solid–liquid extraction technology. Solid–liquid extractions are typically achieved by simple cooking (heating a suspension of biomass in excess solvent for a limited time), Soxhlet extraction (biomass is exhaustively extracted in excess solvent maintained at the boiling point of the solvent for extended period), accelerated solvent extraction, assisted extraction (microwave or ultrasound), supercritical fluid extraction, or extraction at elevated conditions of temperature and pressure (Jablonsky et al. 2015). Both hydrophilic and lipophilic (or hydrophobic) compounds exist in tree bark, which needs to be isolated sequentially with extractants that have similar polarities. Fats, fatty acids, resin acids, sterols, and terpenoids are lipophilic extractives obtained by extractions with non-polar solvents. However, for the extraction of hydrophilic extractives such as carbohydrates, lignans, and phenolic compounds, solvents such as water, acetone, methanol, and ethanol are excellent choices (Dönmez & Önem 2023). Additionally, non-conventional fluids such as ionic liquids, supercritical liquids, and deep eutectic solvents have also been used to produce bark extracts (Feng et al. 2013; Patel et al. 2024).

Solid–liquid extraction is a multi-parameter dependent process with the type of the solvent used, sample/solvent ratio, temperature, extraction time, stirring, type of extraction, particle size, and composition of raw material used affecting the outcome of the process (da Silva et al. 2016; Ferreira-Santos et al. 2020; Supriyadi et al. 2025). Furthermore, sequential extractions isolate products with similar polarities to the extractant used. Therefore, it is likely that the order in which sequential extractions are to be performed has not been investigated as an influential parameter. Most of the research so far has only examined extraction strategies where the bark was sequentially extracted with two to four solvents with increasing polarity (Hafizoğlu et al. 2002; Sandoval-Rivas et al. 2021; Willför et al. 2009). However, to the best of the authors’ knowledge, no studies have been performed investigating the research question – “*will the order of sequential extractions influence the outcome of the process?”.*

The goal of this work was to increase the total yield of a sequential solvent extraction process for *Pinus radiata* bark and to subsequently characterise the resulting extractives. To achieve this, a new parameter—the order in which the solvents are used was examined. In addition to this parameter, the effect of the particle size on the final yield of the extraction process was also examined. Other operational parameters such as solvent to bark ratio, solvent type, extraction time were maintained constant. To test if this parameter would affect the outcome of the biorefinery process, n-hexane was chosen as the non-polar solvent to extract lipophilic extractives, and water was selected as the polar solvent to extract hydrophilic extractives. Finally, a series of analyses were done on the extracts and the bark residue to check whether the change in the order of extractions led to a change in the composition and individual yields.

## Materials & methods

### Materials

All materials and chemicals were used as purchased or obtained until stated otherwise. Technical grade hexane for lipophilic extractions was obtained from Merck Sigma-Aldrich, New Zealand. Water for hydrophilic extractions was produced on-site using a reverse-osmosis system. For gas chromatographic analyses, analytical grade chloroform was obtained from Merck-Millipore, Germany. For the assay of total phenolics (TP) in the extracts, Folin-Ciocalteu (FC) reagent was purchased from Sigma Aldrich, Switzerland), sodium carbonate (Bio-Xtra ≥ 99%) was purchased from Sigma Aldrich, Japan), absolute ethanol was bought from Merck, Germany, ( +) catechin hydrate was bought from Fluka, Indonesia, and polystyrene cuvettes were purchased from Greiner bio-one, Austria. For High Performance Liquid Chromatography (HPLC) analyses, analytical grade methanol was obtained from Supelco, Germany, formic acid was obtained from Thermo-Fisher Scientific, Germany, analytical grade acetonitrile was obtained from Merck, Germany. Epicatechin, epigallocatechin gallate, epicatechin gallate, taxifolin, quercetin standards were obtained from Sigma-Aldrich, New Zealand. For gel permeation chromatography analyses, 0.05 mol/L of lithium chloride (LiCl) in analytical grade dimethyl sulfoxide (DMSO AR) was purchased from Merck, Germany. 

### Bark sourcing and characterization

Two sets of *P. radiata* bark were obtained in its milled form from East Bay Bark Company (Kawerau, New Zealand) and used as received in this work: B1 (particle size less than 1 mm) and B2 (particle size less than 2 mm). The purchased bark was stored at 4 °C until further use. Since the bark was obtained from a commercial supplier, the age and origin of the bark were not known.

The particle size distribution (PSD) of both sets of bark used in this work was determined using the standard method EN 17827–1:2016 (International Organization of Standards [21]) on a vibrating sieve shaker (Humboldt H-4330, ¼ HP motor, Germany) operated with seven sieves of sizes: 4, 2, 1, 0.5, 0.25, 0.125, and 0.063 mm. The bottom most attachment was a pan to collect particles smaller than 0.063 mm. The PSD tests were done in duplicate, and all sieves were cleaned and weighed (tare weight) on a laboratory scale (AND GP-12 K, New Zealand) prior to the start of each test. Prior to any extraction, the moisture content (MC) of a representative sample of the bark used was determined (in triplicate) by weight loss method during oven drying at 104 °C for 24 h (ASTM 2020). 

MC was calculated using Eq. 1:

1 $$\begin{array}{c}Moisture content \left(mass \%\right)=\left( \frac{{{W}_{wet bark}\left(g\right)- W}_{dry bark }(g)}{{W}_{wet bark} (g)}\right) \times 100 \end{array}$$

where W_dry bark_ is the weight of dry bark post 104 °C incubation, and W_wet bark_ is the weight of bark prior to drying.

### Extraction of *P. radiata* bark

**Fig. 1:**
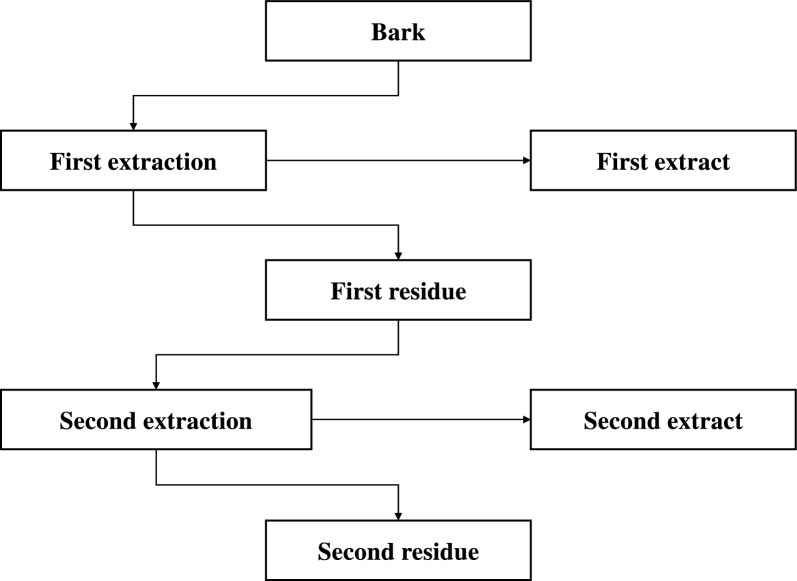
Material flow for the order of extractions of *P. radiata* bark (right)

The sequential extraction of bark was tested with two extractants: hexane (see Sect. "Hexane extraction trials") and water (see Sect. "Hot water (HW) extraction trials"). In total, four trials were performed by altering two variables (extraction order and bark particle size) as depicted in Fig. 1. Not less than three replicates were performed for each trial (Table 1). Identical conditions were used for trials 1 and 3, and 2 and 4, respectively, the only exception being the PSD of the bark used.

**Table 1:** Order of extraction trial matrix (left) with the number of replicates for each trial

Bark size	Trial number	First extractant	Second extractant
B2	1	Hot water (6 replicates)	Hexane (12 replicates)
	2	Hexane (10 replicates)	Hot water (3 replicates)
B1	3	Hot water (3 replicates)	Hexane (3 replicates)
	4	Hexane (10 replicates)	Hot water (3 replicates)

#### Hexane extraction trials

Previous accounts of hexane extractions of different bark species have used the Soxhlet method in a time range of 12–20 h (Autor et al. 2022; Dönmez & Önem 2023; Hafizoğlu et al. 2002). This work is a modification of the reported Soxhlet methods, wherein hexane extraction of *P. radiata* bark (either B1 or B2) was performed by adding 10 g of bark into 250 mL round bottomed flask fitted with a reflux condenser. To this setup, 150 mL of hexane was added, making the ratio 15–1 (extractant to bark), and the suspension was heated to reflux at 70 °C without mixing, for a period of 16 h. At the end of the incubation period, a solid–liquid separation step (gravity filtration) was performed with Whatman filter paper (Grade 1, qualitative, 18.5 cm). The solid residue in the filter paper was washed with an additional 80 mL of hexane. The residue was then air-dried at room temperature for 1 week, after which the residue was weighed again for yield calculations. The hexane in the pooled extracts (liquid fraction, known as the lipophilic extract) of the process was concentrated to dryness by rotary evaporation (Heidolph Heizbad Hei-Vap, Germany) to yield a waxy solid (hexane extract (HE)). The procedure for extractions with hexane as the second extractant was the same with only one difference—air-dried B2 and B1 bark residue from hot water extractions were used instead of fresh bark.

#### Hot water (HW) extraction trials

HW extractions were performed at the ratio of HW to bark (10–1 (volume to mass)) and the temperature of extraction (90 °C) exactly as reported previously (Bridson et al. 2024; Santos et al. 2023). Briefly:

- For trials 1 and 3, where HW was the first extractant: 50 g of *P. radiata* bark was added to a 600 mL glass beaker with 500 mL of deionised water (bark to water ratio: 1:10 w/w) incubated on a hot plate (Heidolph, MR Hei-Tec, Germany).
- For trials 2 and 4, where HW was the second extractant: 10 g of pooled hexane extracted *P. radiata* bark residue was added to 150 mL glass beaker with 100 mL of deionised water (bark to water ratio: 1:10 w/w) incubated on a hot plate (Heidolph, MR Hei-Tec, Germany)

The suspension was mixed at 500 rpm for a period of 30 min, at the end of which the suspension was vacuum filtered through a pre-heated ceramic filter with a Whatmann filter (Grade 1, qualitative, 18.5 cm) until only residue remained on the filter. The filtrate (hydrophilic extract) was freeze dried (Labconco freezezone 6 plus (Model number: 79434037, 6L), United States of America (USA)) to yield a brown fluffy powder (hot water extract (HWE)). The dried extract was weighed and used for yield calculations (Eq. 2) as well. All samples generated by the extraction methods were analysed using different analytical techniques.

The extraction yield for HE and HWE were calculated using formula (2):

2 $$\begin{array}{c}Extraction yield \left(mass \%\right)=\left(\frac{Weight of dry extract \left(g\right)}{Weight of oven dried bark \left(g\right)}\right) \times 100 \#\end{array}$$

where weight of the dry extract represents dry material weight of solids from the liquid fraction, and weight of oven dried bark represents dry bark used in the extraction process.

### Statistical analyses

Statistical analysis for the yield and particle size was conducted using Minitab version 21.4.1. A two-way analysis of variance (ANOVA) was performed to assess the effects of extraction order and particle size on the response variables: total yield, hot water (HW) yield, and hexane (Hex) yield. Post-hoc pairwise comparisons were conducted using Tukey’s test at a 95% confidence level.

The individual compound data were analysed for statistical significance using R version 4.4.2 (2024–10-31 ucrt) (R-Core-Team 2024) and RStudio version 2024.09.1 + 394 (Posit-team 2024). As these data were non-parametric, the Wilcoxon test was used.

### GC–MS analyses of lipophilic extracts

Hexane extract (1 mg) was dissolved in analytical grade chloroform to make a 1 mg/mL solution and injected (1 µL) with a split ratio of 10 to 1 into an Agilent 7890B gas chromatograph fitted with an Agilent J & W Ultra 2 column (50 m × 200 μm × 0.033 μm) coupled to a 5977 B single quadrupole mass spectrometer by an autosampler unit. The carrier gas for these analyses was high purity helium (99.99%) at a flow rate of 1 mL/min with an injection temperature of 280 °C and an interface temperature of 300 °C. The temperature of the column was initially 40 °C and ramped up at 6 °C/min to 300 °C and held at this temperature for 30 min. The conditions for MS were: source temperature (250 °C), quadrupole (150 °C), and ionisation energy (70 mV). Agilent MassHunter Workstation for instrument control and data acquisition software was used for analysing the chromatograms. Initially compounds were found using the identification algorithm in the software and matched against the National Institute of Standards and Technology (NIST) 14 MS spectral library or Scion’s in-house libraries. Results from each compound peak were calculated as relative percentage of the entire area of all the peaks in the chromatogram and it is to be noted that the results do not reflect the relative amounts in the sample, but relative peak areas in the chromatogram.

### Folin-Ciocalteu (FC) assay of total phenolics (TP) in hydrophilic extracts

The TP content in the individual HWE samples was determined as biological replicates (independent samples) using the FC assay (Folin & Ciocalteu 1927; Singleton et al. 1999). Briefly, FC reagent 50 vol. % was prepared fresh prior to use through the 1 to 1 volumetric dilution of FC reagent in double deionised water (ddH_2_O). Sodium carbonate (7.4% (w/v)) was prepared in ddH_2_O. ( +)-catechin hydrate dissolved in 10 (vol.) % absolute ethanol and ddH_2_O mixture was used as the reference standard for this work. ( +)-catechin hydrate solutions of concentration 0.1, 0.05, 0.02, 0.01, 0.005 mg/mL were prepared, and blank measurements were done with 10 vol.% ethanol to water mix.

Around 5 mg of the sample was dissolved and made up to 10 mL using 10% ethanol. The sample solution (0.05 mL) was then added to 0.45 mL water in a test tube while 0.5 mL of the blank and standard solutions were added directly. To the samples and standards, 0.25 mL of the 50% FC reagent and 1.25 mL of sodium carbonate were added and mixed using a vortex. All solutions were allowed to incubate for 40 min before recording UV absorbance was recorded at 750 nm using a UV–VIS Spectrophotometer (Shimadzu UV1800, Japan). The TP content of the HWE were expressed as mg_(catechin equivalent_ _(CE)_ / g_HWE_.

### High-performance liquid chromatography (HPLC) analyses

HWE samples from individual extractions were prepared for HPLC analyses by dissolving 1–3 mg of sample in 1 mL of water/methanol (1:1). Samples (1 μL) were injected into the HPLC instrument (Agilent _1290 HPLC) fitted with a Zorbax Rapid Resolution High Definition (RRHD) SB-C18 (Agilent, United States of America) column (2.1 mmx10 mm, 1.8 μm), with equivalent guard column. Detection of peaks was done on a UV–Vis detector capable of analyses across 190 nm to 400 nm with specific peak detection at 254 nm and 280 nm. ddH_2_O with 0.1% formic acid and acetonitrile with 0.1% formic acid were the mobile phases A and B, respectively. The flow rate used for HPLC analyses in this work was 0.25 mL/min. The gradient for HPLC analyses was as follows: (0 min–10%B, 2 min–10%B, 16 min–90%B, 19 min–90%B, 19.5 min–10%B, 22 min–10%B). The samples were measured as biological replicates, similar to the FC assay.

Individual standards of catechin, taxifolin, and quercetin, were prepared at a concentration of 1 mg/mL in analytical grade methanol as solvent. A combined standard of all these compounds was made with the final concentration of each component reaching 0.1 mg/mL. The results are expressed as μg_(chemical)_/mg_(HWE)_, where chemical represents catechin, taxifolin, and quercetin.

### Gel permeation chromatography (GPC) analyses

Individual HWE samples were dissolved in GPC eluent (see below) to a target concentration of 2.5 mg/mL. Extracts were analysed as biological replicates for molecular weight distribution on a Waters Alliance GPC instrument fitted with a column set containing a guard column and 2 × PSS Gram 100A, 10 µm separation columns (8 mm × 30 mm, PSS, Germany) maintained at 50 °C. Samples were detected by refractive index (RI). An eluent of 0.05 mol/L (M) LiCl in DMSO was used as the solvent at a flow rate of 1 mL/min. Injection volume was 100 µL. An 8-point calibration based on Pullulan standards (PSS, Germany) was used to create a calibration based on peak MW values (180–107,000 g/mol). Samples were evaluated with WinGPC Unichrom (PSS, Germany).

### Proton (^1^H) nuclear magnetic resonance spectroscopy (NMR) analyses

The hot water extract (HWE) and hexane extract (HE) were analysed by proton (^1^H) NMR spectroscopy using a Bruker Avance 400 MHz NMR spectrometer with the sample dissolved in NMR grade dimethyl sulfoxide-d6 (DMSO). Spectral data was obtained in 65 K data points with a relaxation delay of 5 s and 64 scans using the zg30 pulse sequence. Data was assessed using Topspin software (version 3.6.5) for the selection, normalisation, and automatic integration of the peaks (Potrzebowski et al. 1998).

### Fourier transform infrared spectroscopy (FTIR) analyses

FTIR analyses of extracts and residues from both types of extractions were done using a Bruker Tensor 27 instrument with a Bruker Platinum ATR (diamond cell, single bounce). Spectra were acquired with background and sample scans at 4 cm^−1^ resolution from 400 cm^−1^ to 4000 cm^−1^. Spectra were processed with Bruker Opus 8.2 software applying baseline correction (method 2, 64 baseline points), spectral averaging (method weighted with number of scans, compute average report) and normalization (vector normalization from 400 to 4000 cm^−1^).

## Results and discussion

This research was done to investigate if changing solvent order and particle size would influence the individual and total yields of the total extraction process. By using two different solvents in a sequential manner, extractions with HW yielded hydrophilic extracts rich in polyphenols, while hexane yielded lipophilic extracts containing terpenes, resin acids and waxes.

### Total extraction results

**Fig. 2:**
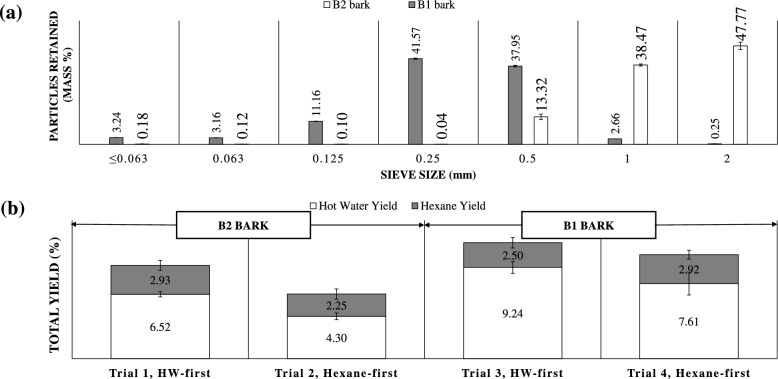
**a** Particle size distribution of two sets, viz. B2 (white) and B1 (grey), of *P. radiata* bark used in the order of extractions work; b Individual yields of pine bark extract by sequential extraction per oven-dried (o.d.) gram of B2 and B1 bark using HW (white) and hexane (grey) as extractants. Error bars are standard deviations of the observations

First, the PSD of bark significantly influences extraction yields, with smaller particles offering higher yields due to greater surface area (Abilleira et al. 2021; Chupin et al. 2015; Ottone & Baldwin 1981; Pătrăuanu et al. 2019) In this study, *P. radiata* bark samples B1 and B2 exhibited distinct PSDs (Fig. 2a). Most B1 particles were between 0.125 and 0.5 mm (91%), with 7% below 0.063 mm, while B2 particles were predominantly within 0.5–2 mm, with only 0.4% smaller than 0.5 mm. Neither sample contained particles above 4 mm. MC of the bark particles used in this work was determined using Eq. 1 and the results are shown in Table 2.

**Table 2:** Moisture content of the bark used for the order of extraction experiments

Bark	Moisture content (weight %)
B1 bark (for HW as first extractant)	12.78 ± 0.10
B1 bark (for hexane as first extractant)	17.30 ± 0.07
HW extracted B1 bark	10.36 ± 0.12
Hexane extracted B1 bark	10.72 ± 0.53
B2 bark (for HW as first extractant)	21.78 ± 0.21
B2 bark (for hexane as first extractant)	21.83 ± 0.20
HW extracted B2 bark	11.76 ± 0.12
Hexane extracted B2 bark	12.09 ± 0.10

Sequential extraction trials (Fig. 2b) demonstrated higher total yields for B1, attributed to its smaller average particle size. Extraction order also affected yields significantly. When hot water (HW) was used first, total yields were higher (9.45% for B2 in trial 1 and 11.74% for B1 in trial 3) compared to when hexane (HE) was used first (6.55% for B1 in trial 2 and 10.53% for B2 in trial 4). These variations were largely driven by differences in HW extraction yields, which showed significant dependence on extraction order (p = 0.0003) and particle size (p = 0.0000). HE extraction yields, ranging from 2.25 to 2.90%, were unaffected by either factor (p > 0.3). Furthermore, total yields calculated as a sum of hexane yield and HW, significantly depended on particle size (p = 0.0001) and extraction order (p = 0.0013). The exact reason for an increased yield due to a change in the extraction solvent was surprising, unknown, and needs to be explored. The authors hypothesize that lignin and carbohydrates were unaccounted in the extracts and would need to be investigated in future work.

This study uniquely investigates the combined effects of particle size, extraction order, and extractant type on total extraction yield, addressing a gap in existing research. Previous studies have focused on sequential solvent use and processing parameters, but did not explore the influence of extraction order. For example, yellow poplar bark studies reported yield increases from 10 to 25% with decreasing particle size, (Ottone & Baldwin 1981) while *P. pinaster* bark yielded 9.9% in hot water extraction for particles smaller than 1 mm (Chupin et al. 2015) These results align with the current findings, emphasizing the importance of particle size in maximizing yields.

A notable finding from the literature review (Table 3) is the dominance of extraction sequences starting with lipophilic solvents, followed by hydrophilic ones, except for the study conducted by Sandoval-Rivas et al*.,* who reversed the order (Sandoval-Rivas et al. 2021) In their study, *P. radiata* bark (0.425–0.5 mm) was first extracted with hot water, yielding 3.12% wax with petroleum ether as the second solvent, compared to 1.2% with ethyl acetate. This behaviour is consistent with waxes' chemical composition, primarily long-chain fatty acids (C16–C24), which are better extracted by the more lipophilic petroleum ether. However, total extractive yields were not reported, limiting direct comparisons with the present study. The findings underscore the importance of optimizing particle size and solvent sequence in sequential extraction processes. Smaller particle sizes enhance yield by increasing surface area for solvent interaction, while starting with hydrophilic solvents like hot water may improve total yields. This work contributes to refining bark-based biorefinery processes by identifying key parameters for efficient extraction and resource utilization. Future studies should explore broader solvent systems and extraction orders to further optimize yields and assess scalability.

**Table 3:** Comparison with earlier studies on the effects of particle size and order of extractions on different biomass using various solvents

Solvent system	Parameters (Solvent to bark, temperature, pressure, time)	Bark information	Total Yield (%)	References
Ethyl ether/ethanol–benzene/cold water/hot water	Ethyl ether/ethanol–benzene: 40 to 1 Cold water: 150 to 1 Hot water: 50 to 1 no other information	Whole, inner, and outer bark of *Liriodendron tulipifer* L. (yellow poplar) < 0.29 mm—> 1.03 mm	10% (> 1.03 mm)	(Ottone & Baldwin 1981)
			13% (0.52–1.03 mm)	
			18% (0.29–0.52 mm)	
			20% (0.14–0.29 mm)	
			25% (< 0.29 mm)	
Chloroform/methanol/water	3 to 1, no info, 1 atm., 2 min	*P. radiata,* (0.425–0.5 mm),	3.27 ± 0.39 Data provided as total wax yield. Individual data at each stage not provided	(Sandoval-Rivas et al. 2021)
1 M sodium hydroxide solution followed by incubation in petroleum ether and ethanol	6.7 to 1, 120 °C, 1.2 atm, 80 min		3.53 ± 0.80	(Sandoval-Rivas et al. 2021)
Ethyl acetate solvent	6.7 to 1, 30 °C, 1 atm., 24 h		3.23 ± 0.21	
Water followed by incubation in petroleum ether	6.7 to 1, 120 °C, 1.2 atm., 80 min		3.12 ± 0.49	
Supercritical carbon dioxide (scCO_2_)/ionic liquid (IL) (cholinium hexanoate)	scCO_2_: 2.7 to 1, 60 °C, 3.5 h IL: 10 to 1, 100 °C, 2–4 h	*P. radiata* bark For scCO_2_: (less than 2 mm) For IL: 0.5 mm	5.2 ± 0.06/2.25 (suberin specific)	(Bento et al., 2022)
Hexane/ethanol/HW (3x)	5 g bark, Soxhlet conditions, no info on temperature, pressure, duration	*Abies bornmülleriana* (< 0.7 mm)	14.6 (5.2/3.4/6.0)	(Hafizoğlu et al. 2002)
		*Castanea sativa (*< *0.7 mm)*	28.1 (2.6/17.4/8.0)	
		*Pinus nigra (*< *0.7 mm)*	43.8 (3.3/38.4/2.2)	
n-hexane/acetone:water (95%v v^−1^)	200 g (10–20 mesh particle size), Soxhlet extraction, no info on temperature, pressure, or time	*Abies pindrow*	13/125 (mg g^−1^ dry bark)	(Willför et al. 2009)
		*Cedrus deodara*	45/95 (mg g^−1^ dry bark)	
		*Pinus geradiana*	85/150 (mg g^−1^ dry bark)	
		*Pinus roxburghii*	18/100 (mg g^−1^ dry bark)	
		*Pinus wallichiana*	60/290 (mg g^−1^ dry bark)	
		*Taxus fauna*	13/120 (mg g^−1^ dry bark)	
Hexane/ethyl acetate/ethanol:water (40% v v^−1^)	No info, 90 °C, 105 atm. (nitrogen), 20 min	*Alnus gelatinosa (Less than 2 mm)*	2.5/11.0/16.4	(Arshanitsa et al., 2022)
Hexane/dichloromethane/ethyl acetate/methanol/water	12.5 to 1, no info, 24 h	*Populus salicaceae (less than 0.2 mm)*	2.1/1.1/2.8/15.6/2.6	(Autor et al. 2022)
scCO_2_/ ultrasound extracted extraction (water/water:ethanol(50:50)/ethanol)	scCO_2_: no info on bark loading, 40 °C, 150 bar, no info on time of extraction Water/water:ethanol(50:50)/ethanol extractions: 12.5 to 1, 200 W, 30 min,	*Pinus abies (*≤ *40 mesh)*	13.66 (5.00/2.78/3.98/1.90)	(Sut et al., 2022)
scCO_2_/ microwave assisted extraction (water/water:ethanol(50:50)/ethanol)	scCO_2_: no info on bark loading, 40 °C, 150 bar, Water/water:ethanol(50:50)/ethanol extractions: 200 W, 50 °C, 18 min,	*Pinus abies (*≤ *40 mesh)*	16.17 (5.00/1.99/4.79/4.39)	
scCO2 / maceration (water/water:ethanol(50:50)/ethanol)	scCO_2_: no info on bark loading, 40 °C, 150 bar, no info on time of extraction Water/water:ethanol (50:50)/ethanol extractions: 12.5 to 1, 25 °C, 30 min	*Pinus abies (*≤ *40 mesh)*	14.39 (5.00/2.63/4.57/2.19)	
Hexane/HW	Hexane: 15 to 1, 70 °C, 1 atm., 16 h HW: 10 to 1, 90 °C, 1 atm., 0.5 h	*P. radiata (0.5–2.0 mm)*	2.25 ± 0.52/4.30 ± 0.34	(This work)
		*P.radiata (finer than 0.063–2 mm)*	2.92 ± 0.44/7.61 ± 1.16	
HW/hexane		*P. radiata (0.5–2.0 mm)*	6.52 ± 0.26/2.93 ± 0.50	
		*P.radiata (finer than 0.063–2 mm)*	9.24 ± 0.60/20.50 ± 0.53	

### Comparative analysis of hydrophilic and lipophilic extracts

This study examined how extraction order and particle size influence the composition of *P. radiata* bark extracts. Lipophilic extracts were analysed by GC–MS, while hydrophilic extracts were examined by FC assay, HPLC, GPC, ^1^H-NMR, and FT-IR.

**Fig. 3:**
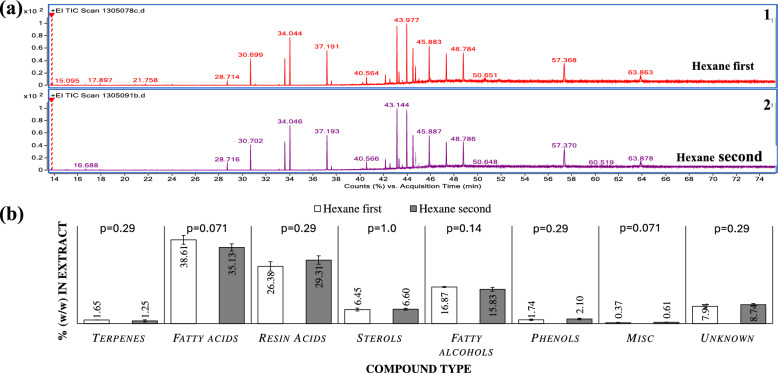
**a** Sample GC–MS chromatogram of lipophilic extracts with hexane as first extractant (top) and second extractant (bottom). **b** Percentage of individual compounds in the lipophilic extracts when hexane was the first extractant (white) (n = 2 replicates) and second extractant (grey) (n = 6 replicates). Error bars are represented as standard deviations and statistical significance (p values) of the data for each class of compounds was tested using the Wilcoxon test

GC–MS chromatograms (Fig. 3a) of lipophilic extracts from hexane-first and hexane-second extractions of B2 bark showed no significant differences in peak profiles, indicating that extraction order did not introduce additional compounds. Additionally, Table 4 lists the individual compounds that were identified in the lipophilic extracts where hexane was the first and second extractant. Major compound classes included terpenes, fatty acids, resin acids, sterols, fatty alcohols, and phenolics, with only minor variations in compound percentages (Fig. 3b). Notably, a slight increase in terpenes was observed when hexane was the second extractant. Unlike previous studies, (Barbini et al. 2021) triglycerides were not detected, likely due to differences in GC–MS temperature settings.

**Table 4:** List of identified individual compounds present in the lipophilic extracts with hexane as first extractant (column 5, from left) and second extractant (column 4, from left) and their relative abundances with respect to the peak integration area

Compound Name	Type	RT (min)	Score (Lib) (roughly)	HW/Hexane	Hexane/HW
Dehydroabietic acid	RA	43.1	86	***22.5***	***20.7***
Lignoceric acid	FA	48.8	79	***13.1***	***14.9***
Behenic acid	FA	45.9	84	***13.6***	***14.5***
1-Tetracosanol	Fal	47.3	81	***11.0***	***11.7***
Beta. Sitosterol	St	63.9	80	***6.6***	***6.5***
Docosanol	Fal	44.7	77	***4.8***	***5.2***
Eicosanoic acid	FA	43.3	79	***3.7***	***3.9***
Pimaric acid	RA	42.2	77	***3.6***	***3.1***
Stearic acid	FA	40.6	85	***2.6***	***2.5***
Isovanillin	Ph	28.7	90	***2.1***	***1.7***
Isopimaric acid	RA	42.5	67	***2.1***	***1.6***
Palmitic Acid	FA	37.6	86	***1.1***	***1.4***
Unknown (45.02 min)	Uk	45.0		1.0	***1.1***
Unknown Resin acid	Uk	46.0		***1.3***	1.0
Unknown (63.31 min)	Uk	63.3		1.0	0.8
Oleic Acid, (Z)-	FA	40.2	57	0.5	0.8
Unknown (45.73 min)	Uk	45.7		1.0	0.7
Hexacosanoic acid	FA	52.6	67	0.5	0.7
Abietic acid	RA	43.6	82	***1.2***	0.6
Unknown (56.32 min)	Uk	56.3		0.5	0.6
.alpha.-Terpineol	T	24.0	90	0.4	0.6
Borneol	T	21.8	74	0.2	0.5
Sandaracopimaric acid	RA	42.4		0.4	0.4
Unknown (46.76 min)	Uk	46.8		0.5	0.4
Unknown (42.76 min)	Uk	42.8		0.2	0.3
Unknown (44.87 min)	Uk	44.9		0.5	0.3
Unknown (44.59 min)	Uk	44.6		0.4	0.3
Unknown (47.62 min)	Uk	47.6		0.1	0.3
Unknown (45.20 min)	Uk	45.2		0.4	0.3
Unknown (48.21 min)	Uk	48.2		0.4	0.3
Bicyclo[3.1.1]hept-2-ene, 3,6,6-trimethyl-	T	13.8	92	0.4	0.3
Unknown Resin acid (40.89 min)	Uk	40.9		0.3	0.3
Ethyl tetracosanoate	M	48.1	66	0.3	0.2
Beta. Pinene	T	15.1	75	0.3	0.2
Unknown (45.40 min)	Uk	45.4		0.2	0.2
Unknown (21.47 min)	Uk	21.5			0.2
Unknown (41.19 min)	Uk	41.2		0.1	0.1
Unknown (40.18 min)	Uk	40.2			0.1
Unknown (45.32 min)	Uk	45.3		0.1	0.1
Methyl dehydroabietate	M	42.6	78	0.3	0.1
.alpha.-Terpineol	T	20.9			0.1
Unknown (39.52 min)	Uk	39.5		0.2	0.1
Unknown (49.35 min)	Uk	49.4	67	0.5	0.1
Unknown (40.33 min)	Uk	40.3			0.1
Unknown (22.77 min)	Uk	22.8			0.1
Vanillic Acid	Ph	33.0	89.88	0.0	
Stearic acid	FA	40.0	67.6	0.0	

*RA *Resin acid, *FA* Fatty acid, *St* Sterol, *Fal* Fatty alcohol, *Uk* Unknown, *T* Terpene, *Ph* Phenolic, *M* Miscellaneous

**Fig. 4:**
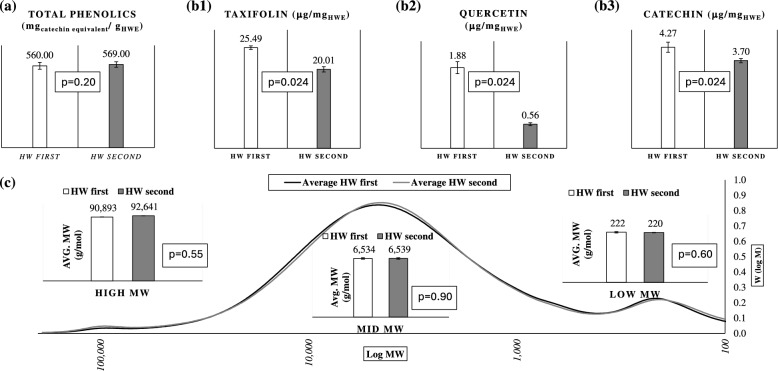
**a** Folin-Ciocalteu assay results of the total phenolics present in HWE of *P. radiata* bark when extracted with HW first (white) (n = 6 replicates) and HW second (grey). (n = 3 replicates). (**b** 1–3) Amounts of taxifolin, quercetin, catechin (in Τμ*gmgHWE*) when extracted with HW first (white) and HW second (grey) (n = 3 replicates). **c** GPC chromatograms of HW first samples (grey) (n = 6) and HW second samples (grey) (n = 3) showing the low MW, mid MW, and high MW region. Error bars shown in the figures are standard deviations of the observations and statistical significance (p values) was examined using Wilcoxon tests

The FC assay (Folin & Ciocalteu 1927; Singleton et al. 1999) assessed total phenolics in the HWE as CE. Regardless of extraction order, the total phenolics content remained consistent: 560 ± 24 mg_CE_/g_HWE_ when HW was the first extractant and 569 ± 19 mg_CE_ /g_HWE_ when HW followed hexane extraction (Fig. 4a). This finding aligns with earlier studies (Ku & Mun 2007; Ku et al. 2011) and suggests that extraction order does not affect the phenolics content of HWE, providing useful insights for designing bark-based biorefineries.

HPLC analysis revealed that the order of extraction significantly impacted specific flavonoid yields (Fig. 4b). Taxifolin content was higher when HW was the first extractant (25.49 µg/mg HWE) than when it was second (20.01 µg/mg HWE). Similarly, quercetin and catechin levels decreased when HW followed hexane extraction. These results were unexpected, as these hydrophilic polyphenols (flavonoids) should not be influenced by prior hexane extraction (Barbini et al. 2021; Ferreira-Santos et al. 2020). Further investigation is needed to clarify this phenomenon.

GPC revealed a trimodal molecular weight distribution in the HWE (Fig. 4c), consistent with previous studies (Ku et al. 2011) Peaks corresponded to high, medium, and low molecular weights. Extraction order had no significant effect on molecular weight distribution, though minor variations were noted. When HW was the first extractant, the average molecular weights were 91,000, 6,500, and 220 g/mol; with HW second, the values were 93,000, 6,500, and 220 g/mol.

**Fig. 5:**
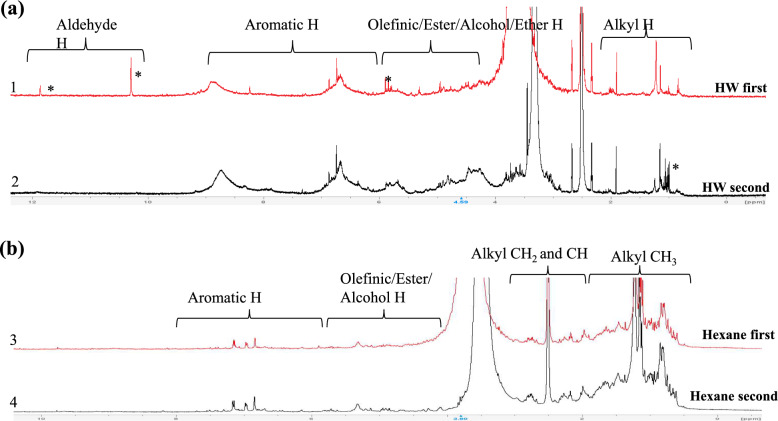
1H NMR spectra of HWE (**a**) and HE (**b**) when water/hexane was the first (red) and second extractant (black)

Proton (^1^H) NMR spectra (Fig. 5) for HW extracts showed subtle differences based on extraction order. Unique peaks at 1 ppm, 4 ppm, 10.5 ppm, and 12 ppm were observed when HW followed hexane. These peaks likely represent residual lipophilic compounds from incomplete washing and drying after hexane extraction. Peaks at 10 ppm and 12 ppm, attributed to aldehydic and carboxylic acid hydrogens (Günther 2013) appeared only when HW was the first extractant, correlating with higher extraction yields in this sequence. Hexane extracts displayed no notable differences in NMR spectra between extraction orders.

**Fig. 6:**
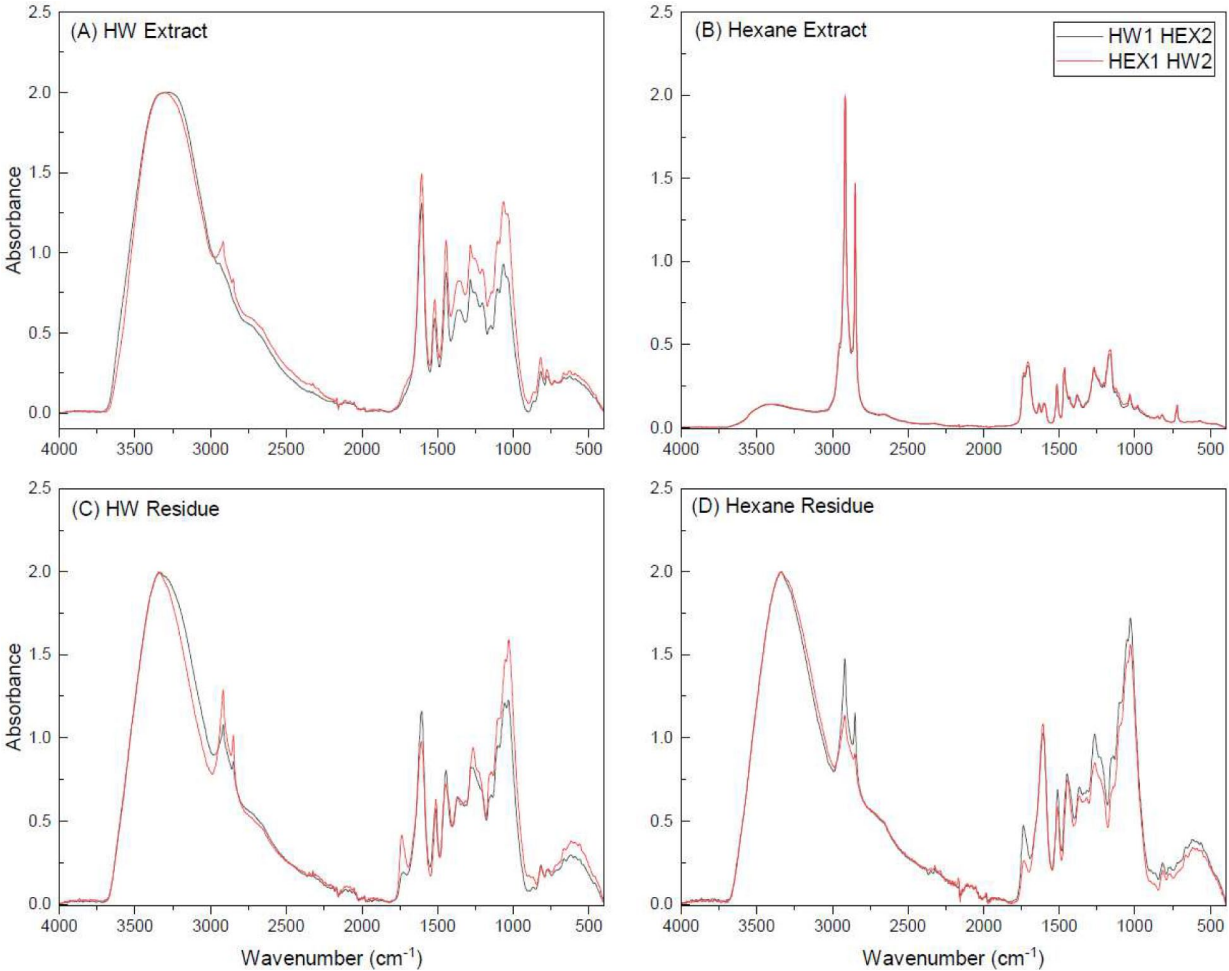
FTIR spectrum of the extracts and residues obtained by the order of extractions work. **A** Hydrophilic extracts when HW was the first extractant (black) and second extractant (red). **B** Lipophilic extracts when hexane was the first extractant (black) and second extractant (red). **c** Hydrophilic residues when HW was the first extractant (black) and second extractant (red). **d** Lipophilic residues when hexane was the first extractant (black) and second extractant (red)

FTIR spectroscopy provided qualitative insights into extract composition (Fig. 6). HWE spectra (Fig. 6a) showed characteristic peaks, including hydroxyl group vibrations at 3300 cm⁻^1^, aromatic carbon–carbon stretching at 1600 cm⁻^1^, and aliphatic C–OH stretching between 1000 and 1100 cm⁻^1^.(Ku & Mun 2007; Mun 2014; Ricci et al. 2015) When HW followed hexane, increased intensities of aliphatic peaks at 2915 cm⁻^1^ and 2850 cm⁻^1^ were noted, suggesting a slightly increased proportion of aliphatic compounds.

Hexane extracts (Fig. 6b) exhibited peaks indicative of saturated aliphatic compounds, including asymmetric and symmetric C-H stretching vibrations at 2920 cm⁻^1^ and 2848 cm⁻^1^. Carbonyl peaks at 1700–1740 cm⁻^1^ were attributed to triglycerides, fatty acids, and resin acids. Extraction order did not significantly alter the FT-IR spectra of hexane extracts. Residue analysis (Fig. 6c, d) showed greater variability in spectral profiles, reflecting the heterogeneous composition of bark. However, the overall profiles of residues from HW and hexane extractions were similar, with minimal extraction altering the remaining material.

### Implications

Extensive characterisation of both lipophilic and hydrophilic extracts shows no significant increase in the type and amount of extracted and analysed compounds. However, a significant increase in the total yield of the two-stage extraction process was observed. The exact reason for this observation is still not clear. One hypothesis is that the higher particle size bark (B2) underwent less shear than the smaller particle size (B1) bark. As already established particle size significantly impacts extraction yields, highlighting the need for size reduction to enhance bioactive compound recovery. The second hypothesis is that *Pinus radiata* bark is a rich source of carbohydrates (5–10%) and lignin (up to 14%), which were not analysed. While extraction order substantially affects total yields, it also influences specific compounds like taxifolin and quercetin, enabling protocol optimization for targeted recovery. The little to no change in phenolic content in hot water extracts, regardless of order, benefits antioxidant-based industries. Consistent molecular weight distribution indicates structural stability, making extracts suitable for biopolymers or functional materials. Residual lipophilic compounds in hydrophilic extracts underscore the need to refine washing and drying steps, improving purity and advancing *P. radiata* bark's potential in sustainable bio-based product development.

## Conclusions

This study successfully establishes the significant effect of bark particle size, and the order of extraction on the hydrophilic (HW) and total yields, while no significant effects were observed for the lipophilic (hexane) yield. Moreover, performing the hydrophilic extraction first would increase the total extraction yield of *P. radiata* bark by 20%. Additionally, a significant increase of the flavonoids catechin, quercetin, and taxifolin was observed, which can be used as antioxidants, antimicrobials, and nutraceuticals. Furthermore, no major differences were observed in the composition of extracts on changing the order of extraction. These insights collectively highlight opportunities to enhance extraction processes, reduce waste, and tailor protocols for specific end-uses, positioning *P. radiata* bark as a valuable resource in sustainable bio-based product development. We recommend that additional tests with other processing parameters such as bark type (other tree species), solvent mixture, and elevated temperature and pressure be examined to confirm the robustness of the process. 

## Availability of data & materials

Data and materials available on request.

## Data Availability

Data is available on request.
